# Personnel scheduling using an integer programming model- an application at Avanti Blue-Nile Hotels

**DOI:** 10.1186/2193-1801-2-333

**Published:** 2013-07-22

**Authors:** Biniyam Asmare Kassa, Anteneh Eshetu Tizazu

**Affiliations:** College of Business & Economics, Bahir Dar University, Bahir Dar, 2052 Ethiopia

**Keywords:** Personnel scheduling, Integer programming, Avanti Blue-Nile Hotels

## Abstract

**Abstract:**

In this paper, we report perhaps a first of its kind application of management science in the Ethiopian hotel industry. Avanti Blue Nile Hotels, a newly established five star hotel in Bahir Dar, is the company for which we developed an integer programming model that determines an optimal weekly shift schedule for the Hotel’s engineering department personnel while satisfying several constraints including weekly rest requirements per employee, rest requirements between working shifts per employee, required number of personnel per shift, and other constraints. The model is implemented on an excel solver routine. The model enables the company’s personnel department management to develop a fair personnel schedule as needed and to effectively utilize personnel resources while satisfying several technical, legal and economic requirements. These encouraging achievements make us optimistic about the gains other Ethiopian organizations can amass by introducing management science approaches in their management planning and decision making systems.

**Electronic supplementary material:**

The online version of this article (doi:10.1186/2193-1801-2-333) contains supplementary material, which is available to authorized users.

## Introduction

Efficient utilization of personnel resources in large organizations requires proper allocation of personnel to keep costs as minimal as possible while meeting several constraints. Personnel scheduling involves several elements including determination of required workforce, allocation of the workforce to duties (Hillier and Lieberman 2001) where the later includes assignment of personnel to shifts and locations. Thus, personnel scheduling deals with providing the right people at the right time at the right cost whilst achieving a high level of employee satisfaction.

On the face of escalating personnel costs especially among service organizations such as airlines, hotels, hospitals and other service organizations the effective planning and allocation of work force is a critical success factor for these organizations (Glover and McMillan 1986). The importance of disciplined and systematic personnel planning approach obtains on this account.

The vast management science literature in the area of personnel scheduling is in accord with the wide importance of effective personnel planning. Several applications of operational research approaches to effectively plan and allocate work force suggest the significant improvements that accrue to organizations that have bowed themselves to the wisdom of systematic and disciplined approach to personnel planning. For instance, Gendron (2005) report an application of web-based integer programming algorithm to develop optimal weekly employee schedule for a Quebec based liquor Stores Company. The application of the OR model helped the company save about 1 million dollars per year. A related application is in class scheduling in universities and schools (see for instance, Dinkel et al. 1989).

Typically, personnel planning based on a management science approach begins with a forecast of number of employees required for each of the periods under consideration. On the basis of service level requirement estimates (using such techniques as regression analysis), a mathematical model may be used to determine cost efficient number of workers that should be hired during a given planning horizon. If necessary, the next stage may include developing a tour-schedule for each employee. Often integer programming and related models and heuristics are used for these purposes (Vanhoucke and Maenhout 2005). As the size of the organization increases and as operating requirements become complex the modeling task may become very difficult. For many large organizations, the gains in cost savings as well as personnel satisfaction from applying such disciplined approach to personnel scheduling is worthy of the effort. While experience from many companies suggests that companies are increasingly using a management science approach to personnel planning, a glimpse at local practices suggests many organizations both private and public are paying a heavy toll in terms of excess labor force to compensate for the lack of systematic management. This, for sure, is self-defeating in the long term. With the increasing number of professionally trained managers in Ethiopian organizations, a new direction that improves the current state of the art of personnel planning is undoubtedly recommendable. We expect that the application reported in this paper will help inspire local managers to find interest in the use of management science approaches to improve decision making in personnel planning as well as other areas.

## The problem

The problem addressed in this report regards the assignment of five personnel in the engineering department of Avanti Blue Nile Hotels, Bahir Dar, Ethiopia across three shifts in a seven days week. Avanti Blue Nile Hotel is a newly established Hotel officially starting operation only as of June 2012.

The hotel’s personnel manager who was aware of the difficulty of developing a sound tour-schedule in the absence of some support from a management science approach called up on our assistance in the exercise. We accepted the invitation to assist him in developing the required schedule. The challenge facing the Hotel’s personnel management is the assignment of five employees in the engineering department across three daily shifts in a seven days week. The problem is a difficult combinatorial one. In the presence of several sets of internal and external constraints, developing a schedule for each employee that satisfies all the constraints is even more difficult. Given the small number of personnel to assign, we found it efficient to address it in a holistic manner using a binary integer programming model that can be easily implemented on an excel solver routine.

In the problem we modeled, there are three shifts per day- the morning shift (between 8 AM and 4 PM), the afternoon shift (between 4 PM and 12 AM) and the night shift (between 12 AM and 8 AM). Each shift has 8-hour duration, and the same starting and ending time in each day of the week.

Assignment of the five personnel ought to be done while satisfying different requirements. Two general requirements in the model limit workload and shift sequence allocation to each employee. An employee cannot be on duty for more than 48 hours each week. In addition, each worker must have 16 consecutive rest hours before and after any work shift assigned to him/her, and each employee should get 24 consecutive rest hours per week.

Another set of constraints regards personnel requirement per shift. There should be at least some number of employees at work in each of the shifts in a week. Ideally, this should be based on estimated personnel requirements, which may vary from shift to shift and from day to day. However, the personnel management told us that the demands differ little from shift to shift mainly because the engineering department’s work volume is not significantly related with the hotel’s main activities. For this reason, a simple requirement that at least one personnel be available in each shift was adequate.

Another requirement is that each employee should get six work shifts per week or else the employee’s case is considered separately. In addition, assignment of employees to different shifts in the week should be as fair as possible. For instance, if an employee is assigned to six night shifts out of the seven night shifts in a week, but the remaining employees are assigned one or no night shifts, there is clearly a lack of fairness in the assignment, given that employees may less prefer night shifts.

## An integer program formulation

We formulated a binary integer program model that determines a weekly tour-schedule for the five employees of the engineering department while maximizing work done and satisfying a set of constraints described in this section.

### Notations

We denote an employee by *j*where j= 1,2,3,4 & 5 is the complete list of the five employees of the department. In the model, employees are not identified by names, because there are no employee specific considerations whatsoever. However, it is possible to formulate the model taking individual-specific considerations such as seniority, sex and so on into account. Nevertheless, the personnel manager told us that there are no important individual specific considerations for the time being.

We denote a shift in a week by *i*where *i*= 1, 2,….21 is the complete list of all shifts in a week. There are three shifts in each of the seven days of the week. We call these shifts the morning, the afternoon and the night shifts. The morning shift is between 8 AM and 4 PM. The afternoon shift is between 4 PM and 12 AM. The night shift is between 12 AM and 8 AM. There are 21 shifts in a seven-day week. For convenience, we take Monday morning as first shift and consecutively count the next shifts in the week up to 21.^a^ Hence, we use the subscripts (1, 4,7,10,13,16 and 19) to refer to the morning shifts; the subscripts (2,5,8,11,14,17 and 20) to refer to the afternoon shifts and subscripts (3,6,9,12,15,18,21) to refer to the night shifts.

### Variables

#### *Employee-shift match indicator variables*

To indicate whether a worker is assigned to a given shift we use the following binary variables: 

There are 21 variables per employee. Since there are five employees, there are 105 employee-shift matching variables. These variables are binary (either 0: that shift *i* is not assigned to employee *j*; or *1*: that shift *i* is assigned to employee *j*).

#### *Variables indicating inclusion/exclusion in weekly schedule*

Suppose there is no feasible schedule such that all requirements are satisfied and an employee is retained in the final schedule. If such possibility arises, it is necessary either to change other constraints or to allow the possibility of excluding an employee from the schedule. Since the latter is the more general way, we introduced the binary variables *y*_*j*_ for employee *j*that indicate whether employee *j* is included in the final schedule. 

If *y*_*j*_ = 1 employee *j* is included in the final schedule, if *y*_*j*_ = 0, the employee is excluded from the schedule. There are five of these variables in our model, one per employee.

### Objective function

The objective was to maximize the number of personnel who are at work per week. 1

As noted above, *y*_*j*_ is the binary variable indicating whether an employee is included in the final schedule while satisfying all the remaining constraints. If an employee is included in the optimal schedule, it means the employee has been assigned six work shifts per week. Since the Hotel has already decided the required number of personnel, the possibility of minimizing personnel cost, a common objective in this kind of problem, is not relevant. Given there are five employees already hired, the other acceptable criterion of effectiveness is to maximize the utilization of these personnel. Hence, the objective function of maximizing number of personnel assigned full time work is taken as the criterion of effectiveness.

### Constraints

#### *Constraints on rest hours before and after an assigned work shift*

For each employee *j*, it is required that the gap between one shift assignment and the next be at least two shifts. Specifying minimum rest time between working shifts is a common practice in most personnel scheduling problems (Cheang et al. 2003). For example, if an employee is on duty on Monday morning shift, the employee must not be on duty before the next Tuesday morning shift and after the previous Sunday morning shift. Thus, an employee can be on duty on a given shift only after two rest period shifts from his/her previous duty shift and his/her next duty shift can’t occur before two rest periods after his/her current duty shift. These requirements are converted into a set of inequality constraints. 2

Note that the first set of constraints and the last two sets are similar in nature. However, the *k+1* and *k+2* terms when *k=21* are *i=1* (the next week’s Monday morning shift) and *i=2* (the next week’s Monday afternoon shift) which is not convenient to maintain the consistency of the indexation used here. Putting these inequalities separately also makes it clear that next week assignments are also included in satisfying the rest hour requirement between shifts (full cycle of assignment shifts are considered). There are 21 constraints per employee making 105 constraints for the five employees.

### Personnel requirement per shift

The personnel manager of the company told us that at least one employee should be available in each shift. These requirements are specified as follows: 3

There are twenty-one of such constraints one for each of the 21 shifts.

### Limit on night shift assignments per employee

Conceivably not all shifts are equally desirable for the employees. In light of this, asking employees to reveal their shift preferences and incorporating these preferences into the model may be suggested as one option (Warner 1976).^b^ However, considering the fact that there are no pay differentials depending on work shift, employees may inflate their preferences and it may be unfair in the end to assign them based on preferences. Consulting with the personnel manager, we continued by assuming equal weight to all employees’ shift preferences. Nonetheless, it is reasonable to assume that night shifts are less desirable. Nevertheless, without additional constraints, there is no guarantee that our model will not assign one employee all night shifts. This will be inequitable in view of the employee who works for many more night shifts than other shifts. Therefore, to avoid these possibilities, we included constraints that set maximum number of night shifts relative to number of other shifts assigned to each employee. Specifically, we restricted that the number of night shifts assigned to an employee be no more than the number of morning as well as afternoon shifts. These give rise to ten additional constraints. 4

5

Note here that the shift indicator subscripts (3, 6, 9, 12, 15, 18, and 21) refer to the night shifts.

### Weekly total number of shifts per worker

Another constraint is the number of hours worked per week by each employee. Each employee is required to work only for 48 hours (which means six eight-hour shifts per week). If an employee can’t be feasibly assigned to six shifts per week, the employee should be excluded from the current model. Given the variables that indicate whether an employee is included in the optimal weekly tour-schedule, the workload constraints were converted into the following equations. 6

There are five constraints of this type in total, one per employee.

In summary, the model included 141 functional constraints (excluding the constraints directly imposed on the variables) and 110 decision variables.^c^

The model developed to solve the personnel tour-scheduling problem is summarized as follows:

### Model formulation



 Subject to; 

### Implementation

We solved the binary integer model using Excel solver routines on a personal laptop computer. In spite of the large number of binary variables and constraints, solving the model took us less than four minutes.

An interesting feature of the model is that there are alternate optimal solutions. Since the shifts are differentiated little with each other (except for the night shift allocation constraints), and since employees are considered as homogenous, this result is not surprising. If there is an optimal solution for given indices of workers one can reshuffle the indices and obtain another schedule as optimal as the original one. Tightening the model by including such restrictions as precise personnel demand for each shift based on real time data, setting upper bounds for the number of personnel per shift and so on, one may expect a unique optimal solution. In its own right, the presence of alternate optimal solutions is desirable. The rationale is it affords management to achieve the same result for the main objective, allowing for additional qualitative considerations in making ultimate choice.

Table 1 shows one solution for the problem. Table 2 provides another condensed summary of the solution. Based on this optimal solution, the number of employee-shifts assigned to morning, afternoon and night shifts are 10, 12 and 8 employees per week respectively. Employee 1, 2 and 5 are assigned equal number of morning, afternoon and night shifts (2 assignments on each shift) while employee 3 is assigned 3 morning shifts, 2 afternoon shifts and 1 night shift and employee 4 is assigned 1 morning shift, 4 afternoon shifts, and 1 night shift. If we continue to assume the undesirability of the night shift, this assignment still appears to be more or less fair in that employee 4 is assigned only 1 night shift and 4 afternoon shifts (assume the afternoon shift is less desirable than the morning shift).

**Table 1:** **An optimal schedule**

Shift ID			Employee ID					Number of
								employees
								assigned
	**Day**	**Shift**	**1**	**2**	**3**	**4**	**5**	
1	Monday	Morning	1		1			2
2		Afternoon				1		1
3		Night					1	1
4	Tuesday	Morning		1				1
5		Afternoon	1		1	1		3
6		Night					1	1
7	Wednesday	Morning		1				1
8		Afternoon	1		1			2
9		Night				1		1
10	Thursday	Morning					1	1
11		Afternoon		1				1
12		Night	1		1			2
13	Friday	Morning				1	1	2
14		Afternoon		1				1
15		Night	1					1
16	Saturday	Morning			1			1
17		Afternoon				1	1	2
18		Night		1				1
19	Sunday	Morning	1		1			2
20		Afternoon				1	1	2
21		Night		1				1
No of shifts assigned			6	6	6	6	6

**Table 2:** **Summary of optimal schedule**

Employee ID	1	2	3	4	5	Total
**Shift**						
Morning	2	2	3	1	2	10
Afternoon	2	2	2	4	2	12
Night	2	2	1	1	2	8

All employees are assigned six shifts per week (48 hours of working time) and each of them is given a weekly 24 hours of consecutive rest period^d^; both of which are required in the problem. All the employees are fully utilized given the weekly workload bounds to be satisfied. Table 3 summarizes our Integer programming model.

**Table 3:** **Summary of model components**

Model component	Sub components	Description	Notes
**Objective function**	There is a single objective function to be maximized	Maximize number of personnel work performed per week measured by number of employees included in the plan	One objective function equation is included
**Constraints**	Shift sequence restrictions	No worker is to be assigned more than one shift out of three consecutive shifts	For each employee there are 21 inequality constraints
	Personnel requirement per shift	At least 1 personnel is required in each shift	There are 21 inequality constraints, the same as the number of shifts in a week
	Night shifts per employee	Limits the number of night shifts an employee should serve relative to other shifts the employee is at work. The weekly number of night shifts an employee is on duty should not exceed the number of morning shifts as well as the number of afternoon shifts.	There are 10 constraints in total, 2 per employee.
	Weekly total number of shifts per worker	The number of shifts an employee should work in a week is specified by personnel department policy to be exactly 6 shifts. If an employee can’t be assigned to 6 shifts per week the employee is excluded from the assignment plan.	There are equations indicating that each personnel should work exactly 6 shifts per week, or none otherwise.
**Decision variables**	Employee-shift matching variables	The yes or no response variables that indicate whether an employee is assigned to a given shift *Xij=1* if shift *i* assigned to employee *j*.	There are 21 variables per worker, and a total of 105 variables
	Variables indicating inclusion in weekly schedule	*y*_*j*_=1 if employee j is included to work in the weekly shifts, 0 otherwise	There are 5 variables, one per employee

Other alternative solutions are derived by solving the model starting from the current solution. Two alternative schedules are summarized and presented in the Additional file 1: Appendix.^e^ As results in these tables suggest, there is wide flexibility for the personnel department in designing an optimal schedule. For instance, the result in Additional file 1: Table S1 suggests that it is possible to allocate all employees equal number of all the shifts if fairness is the main issue. Quantifying and incorporating preferences in the objective function can also ensure fairness (Warner 1976). On the other hand, as Additional file 1: Table S2 suggests one can also make a highly asymmetric assignment if other considerations are more important than fairness in terms of shift allocations among employees.

It is also possible to change the indices of shifts, or equivalently the first and last shift can be changed such that the results from the model can be interpreted within the changed shift indices. As such, it is also possible to assign employees to all the different shift patterns turn by turn in a periodic basis, say monthly or bi-monthly. This is another way of ensuring fair allocations of shifts among employees.

## Conclusion

This application makes it clear that with the assistance of OR/MS techniques local enterprises can simplify their management’s task and amass the benefits of effective and fair utilization of their scarce resources. With regard to the specific problem considered in this report, some key points deserve mentioning.

First, for reasonably small number of personnel, we recommend that companies can use Excel solver routine on a personal computer to solve their problems. Nevertheless, even when we introduce additional personnel into our model, the processing capabilities of personal computer faced difficulties to generating solutions quickly. The combinatorial integer programming nature of the problem explains this. Under such circumstances, we think using computer with greater processing capabilities, along with more specialized software packages such as LINDO worth the savings in time and cost. Many organizations such as hotels and hospitals will substantially benefit from using the approach developed here, perhaps with some modifications to account for local differences among the organizations.

Second, we suggest that future applications among local organizations had better start not with specified number of employees, except for some reasonable justifications for retaining existing employees. The more optimal approach is to start with an integer programming model that determines the cost minimizing number of employees that satisfies required number of employees in each shift or day considering demand for services of the organization and other constraints. After the required number of personnel is determined, the personnel–tour scheduling problem considered here can be solved and appropriate tour-schedules for each of the employees can be developed.^f^

In developing tour schedules, it is also possible to include additional considerations for a more satisfying solution. For instance, an organization may give its senior and women employees the right for fewer night shift assignments. This can be done for instance by assigning penalty weights for night shift variables that represent assignment of these employees. In addition, precise estimates of personnel requirements per shift or day may be included for results that are more accurate.

Finally, let us conclude with some general remarks. First and for most, we cannot exaggerate our excitement in seeing the application of the technique that we teach our students to solve such significant real life problems. There is nothing disturbing for a teacher in a professional field such as in management than teaching a subject whose local applications can’t be verified for a full class of students who are expected to make use of what they learnt in class in solving their society’s problems. At least we now have one modest example to show our students. Second, in spite of the fact that operations research/management science techniques are in extensive use in optimal running of all kinds of organizations in the developed world, we are not aware of any such uses among local organizations. The awareness among the business community as well as leaders of public and other not-for-profit institutions is perhaps a major impediment. Lack of expertise in OR/MS is also a key factor. The beginning of collaboration between university and businesses like the one reported here is a good start. We hope that this work could provide the inspiration needed.

## Consent

Written informed consent was obtained from the personnel manager of Avanti Blue-Nile Hotels for the publication of this report and any accompanying information.

## Endnotes

^a^ If we were to use any other day we could still generate an alternative assignment.

^b^ If employees revealed their shift preferences, the objective would have been maximizing total employees’ preferences.

^c^ The full model then incorporates seven groups of components: an objective function, five groups of functional constraints and variable constraints.

^d^ This requirement is not explicitly included in our model because it is implicitly satisfied whenever the sixteen hours rest requirement and the 48 hour weekly load requirements are satisfied.

^e^ Refer to the Additional file 1: Appendix for alternative solutions.

^f^ Especially profit seeking organizations adopt this two-stage scheduling process.

## Electronic supplementary material

Additional file 1: **Appendix.** (DOC 44 KB)
