# Supplementary material for: Personnel scheduling using an integer programming model- an application at Avanti Blue-Nile Hotels
Source: Springerplus. 2013 Jul 22;2:333. doi: 10.1186/2193-1801-2-333 (PMC3923920; doi:10.1186/2193-1801-2-333)
Supplement: Supplementary file 1 — Additional file 1: Appendix. (DOC 44 KB) [file 40064_2013_457_MOESM1_ESM.doc]

Table S1: Summary of an alternate optimal solution with equal number of assignments of each shift to each employee

| Employee ID  Shifts | 1 | 2 | 3 | 4 | 5 | Total |
| --- | --- | --- | --- | --- | --- | --- |
| Morning | 2 | 2 | 2 | 2 | 2 | 10 |
| Afternoon | 2 | 2 | 2 | 2 | 2 | 10 |
| Night | 2 | 2 | 2 | 2 | 2 | 10 |

Table S2: Summary of an alternate optimal solution with asymmetric assignment of employees to different shifts

| Employee ID  Shifts | 1 | 2 | 3 | 4 | 5 | Total |
| --- | --- | --- | --- | --- | --- | --- |
| Morning | 0 | 2 | 2 | 3 | 2 | 9 |
| Afternoon | 6 | 2 | 2 | 2 | 2 | 14 |
| Night | 0 | 2 | 2 | 1 | 2 | 7 |
